# From Antigenic Drive to Clonal Autonomy: An Update on Molecular Mechanisms of HCV-Related B-Cell Lymphomagenesis

**DOI:** 10.3390/cancers18172761

**Published:** 2026-08-25

**Authors:** Silvia Marri, Maria Concetta Scavuzzo, Gabriella Cavallini, Laura Gragnani

**Affiliations:** Department of Translational Research and New Surgical and Medical Technologies, University of Pisa, 56126 Pisa, Italy; silvia.marri@med.unipi.it (S.M.); mariaconcetta.scavuzzo@unipi.it (M.C.S.); gabriella.cavallini@unipi.it (G.C.)

**Keywords:** hepatitis C virus, lymphomagenesis, mixed cryoglobulinemia, B-cell lymphoproliferative disorders, non-Hodgkin’s lymphoma, lymphotropism, microRNAs

## Abstract

Chronic hepatitis C virus (HCV) infection is an established risk factor for B-cell lymphoproliferative disorders and represents a paradigmatic model of infection-driven lymphomagenesis. Although direct-acting antivirals have markedly reduced the burden of HCV-related disease, HCV-associated lymphomas continue to occur. Moreover, HCV screening remains incomplete in some geographical areas and healthcare settings, leaving a substantial proportion of infected individuals unaware of their status. This narrative review integrates current evidence on the mechanisms linking chronic HCV infection to mixed cryoglobulinemia and overt B-cell non-Hodgkin lymphoma. HCV lymphotropism and persistent antigenic stimulation could initially promote the selection and expansion of autoreactive B-cell clones, while mixed cryoglobulinemia represents the most informative pre-lymphomatous risk condition. Cytokine-mediated survival signals, particularly those involving B-cell activating factor, reinforce clonal persistence and cooperate with host genetic susceptibility, impaired apoptotic control, and activation-induced cytidine deaminase-mediated genomic instability. The progressive acquisition of somatic driver mutations, copy-number alterations, and epigenetic and transcriptomic changes may enable selected clones to escape functional anergy and become increasingly independent of the original viral stimulus that, in turn, represents an initial trigger of the lymphoproliferative process. Recurrent abnormalities converge on *NF-κB*, *NOTCH*, chromatin-regulatory, apoptotic, and cell-cycle pathways, although HCV-associated lymphomas remain molecularly heterogeneous. Emerging microRNA profiles further contribute to the molecular characterization of the transition from chronic infection and cryoglobulinemia to lymphoma. Despite the availability of highly effective antiviral therapies, HCV-associated lymphomagenesis remains clinically relevant and continues to provide an especially informative model for understanding how chronic viral infection can drive human cancer development.

## Simple Summary

Chronic hepatitis C virus (HCV) infection is a well-established risk factor for B-cell non-Hodgkin lymphoma and represents one of the best models of infection-driven cancer development. Although the introduction of antiviral therapies has markedly reduced the burden of hepatitis C, lymphomas continue to occur in some infected individuals. This is further complicated by the proportion of people living with undiagnosed HCV infection, particularly in geographical areas where access to screening/healthcare services remains limited. This review summarizes current evidence on the biological processes involved in HCV-associated lymphomagenesis, including viral persistence in lymphoid cells, chronic immune stimulation, cryoglobulinemia, and genetic and epigenetic alterations. By integrating findings from immunology, molecular biology, and hematology, we provide an updated overview of the pathways driving lymphoma development and progression. A better understanding of these mechanisms may facilitate the identification of biomarkers of disease evolution and support the development of more effective management of infection-associated lymphoid malignancies.

## 1. Introduction

Chronic hepatitis C virus (HCV) infection is a condition that extends beyond hepatotropism and can involve lymphoid cells, creating a biologic framework for immune dysregulation, autoimmunity, and B-cell neoplasia. It is well known that a paradigmatic HCV-driven extrahepatic disease is mixed cryoglobulinemia (MC), a benign, pre-lymphomatous B-cell lymphoproliferative disorder characterized by circulating immune complexes containing polyclonal IgGs and mono-/polyclonal IgM with rheumatoid factor (RF) activity [1]. MC may evolve into cryoglobulinemic vasculitis (CV), and symptomatic MC/CV represents a particularly informative model of lymphomagenesis because the risk of non-Hodgkin lymphoma (NHL) is markedly increased in this setting [1,2,3]: in fact, patients with HCV-CV showed a 35 times higher risk of developing lymphoma compared to the general population [4]. Importantly, however, MC should be viewed as a risk state rather than an obligatory intermediate in a deterministic sequence, since the large majority of affected patients never develop lymphoma. Both indolent and high-grade lymphoma subtypes have been described in association with chronic HCV infection; among these, diffuse large B-cell lymphoma (DLBCL) and marginal zone lymphoma (MZL) are the two most frequent [5].

The introduction of direct-acting antivirals (DAAs) more than a decade ago has profoundly changed the epidemiology of chronic HCV infection and its complications, significantly reducing the burden of HCV-related CV and NHL [1]. Nevertheless, and somewhat unexpectedly, despite the well-established association between HCV and NHL and the decline in HCV prevalence following the widespread use of DAAs, a recent study reported that only about half of newly diagnosed DLBCL patients were screened for HCV (4% of those tested were positive) [6]. These findings underscore that HCV-related lymphoma remains a current and potentially underdiagnosed entity, particularly among individuals unaware of their infection [6,7]. Thus, despite the therapeutic revolution brought about by DAAs, HCV-related lymphomagenesis remains a clinically relevant and biologically informative field, as lymphomas continue to be diagnosed in this setting. Moreover, the well-characterized link between chronic viral infection, sustained antigenic stimulation, and B-cell transformation provides a valuable model for understanding the mechanisms underlying other infection-driven malignancies, particularly in the onco-hematologic setting.

Against this background, the present review provides an updated synthesis of HCV-associated lymphomagenesis by integrating classical, but methodologically debated, evidence on possible viral lymphotropism with data on chronic antigenic and inflammatory stimulation and with recent genomic, single-cell, transcriptomic, and microRNA studies. Rather than assuming a single linear MC-to-lymphoma trajectory, we examine how distinct initiating mechanisms may converge on clonal B-cell expansion and how subsequent molecular evolution can generate increasing autonomy from the original viral stimulus. Although highly effective DAAs have reduced HCV prevalence and the burden of related lymphoproliferative disorders in several regions, the problem is likely underestimated because HCV testing remains incomplete in hematological practice, many infected individuals are unaware of their status, and screening and treatment access remain uneven across geographical areas. Thus, HCV-associated lymphoproliferation continues to represent both a clinically relevant complication and an informative model of infection-associated malignant transformation.

The originality of the present review resides in moving beyond a descriptive summary of HCV-associated lymphomagenesis to provide an integrated and critical model of the transition from antigen-dependent B-cell expansion to clonal autonomy, incorporating recent genomic, immunogenetic, transcriptomic, and epigenetic evidence and translating this biological transition into clinically relevant concepts of persistent risk after viral eradication.

## 2. Methods

This article was designed as a narrative review aimed at summarizing and integrating current evidence on the biological mechanisms linking chronic HCV infection to B-cell lymphoproliferative disorders and NHL. The review focused on mechanistic, translational, and clinical studies addressing HCV lymphotropism, mixed cryoglobulinemia, chronic antigenic stimulation, B-cell activation and survival pathways, genetic instability, recurrent driver mutations, and epigenetic (such as microRNA) dysregulation or transcriptomic remodeling in HCV-associated lymphomagenesis.

A literature search was performed in PubMed/MEDLINE covering publications from 1989 to 2026, with the final search conducted on 30 June 2026.

The search strategy combined terms related to HCV infection with terms describing B-cell lymphoproliferation, disease stages, and molecular mechanisms. The principal search string was: (“hepatitis C virus” OR HCV) AND (“B cells” OR “B-cell lymphoproliferative disorders” OR “mixed cryoglobulinemia” OR “cryoglobulinemic vasculitis” OR “non-Hodgkin lymphoma” OR “marginal zone lymphoma” OR “diffuse large B-cell lymphoma”) AND (lymphotropism OR “antigenic stimulation” OR BAFF OR “B-cell activating factor” OR AID OR “activation-induced cytidine deaminase” OR genomics OR genomic OR mutations OR NOTCH OR “NF-kB” OR transcriptomics OR transcriptomic OR “B-cell receptor” OR “immunoglobulin repertoire” OR microRNA OR miRNA OR epigenetics OR “clonal evolution” OR “clonal autonomy”). Additional targeted searches combining individual terms were performed when necessary, and the reference lists of relevant original articles and authoritative reviews were manually screened to identify additional studies.

Eligible publications included original human studies, translational and mechanistic studies, genomic, immunogenetic, transcriptomic and epigenetic analyses, and selected experimental studies providing relevant mechanistic information on HCV-associated B-cell lymphoproliferation. Particular attention was given to studies addressing HCV lymphotropism, chronic antigenic stimulation, MC/CV, B-cell clonal selection and persistence, BAFF-mediated survival, AID-associated genomic instability, recurrent driver alterations, NOTCH and NF-κB signaling, clonal evolution, MZL and DLBCL, and molecular changes persisting after antiviral therapy. Recent high-quality reviews were also considered to contextualize established findings and areas of ongoing controversy. Studies lacking direct relevance to HCV-associated lymphoproliferation, isolated case reports without mechanistic information, and publications for which sufficient methodological or clinical information was unavailable were not prioritized.

Approximately 150 publications were ultimately took into account and 110 were included in the narrative synthesis, encompassing historical studies that established the HCV–lymphoproliferative disease association as well as more recent genomic, transcriptomic and biomarker studies. Because of the narrative nature of the review and the substantial heterogeneity of the included studies with respect to study design, patient populations, biological specimens and analytical methodologies, no formal meta-analysis or quantitative risk-of-bias assessment was performed. Instead, throughout the review, established observations were distinguished from findings considered mechanistic, controversial, or exploratory.

## 3. HCV Lymphotropism: Evidence and Methodological Caveats

Since the 1990s, converging experimental and clinical observations have established that HCV exhibits a lymphotropic propensity in addition to its hepatotropism [8,9,10,11,12,13]. HCV RNA has been detected in peripheral blood mononuclear cells (PBMC), in some cases even in the absence of detectable serum viremia, suggesting that hematopoietic cells may represent an independent viral compartment as well as a viral reservoir [14,15,16,17]. However, detection of genomic HCV RNA in a cellular preparation does not by itself establish productive intracellular infection, because extracellular RNA and cell-associated virions may contaminate PBMC preparations. The first evidence supporting active infection includes the demonstration of increased intracellular viral RNA following mitogenic stimulation, identification of both genomic and replicative (negative-strand) RNA intermediates using strand-specific methodologies, detection of viral proteins within lymphoid cells, and persistence of infected PBMC after transfer into immunodeficient mouse models [11,12,13,18]. These observations are biologically suggestive, but their interpretation depends critically on the specificity of the methods used to demonstrate viral replication.

Nevertheless, for several years, significant methodological concerns were raised regarding the strand-specific detection of the replicative (negative-strand) HCV RNA intermediate, as well as the potential contribution of extracellular viral RNA contamination to false-positive results [19,20]. Early PCR-based approaches were particularly prone to artifacts caused by self-priming and mis-priming events, whereby the abundant positive-strand RNA could generate spurious complementary strands during reverse transcription, thereby confounding the interpretation of viral replication [19]. In addition, the presence of circulating virions adhering to PBMC further complicated the distinction between true intracellular infection and passive viral adsorption [20].

Importantly, the generally low levels of viral replication and the limited proportion of infected cells likely account for the heterogeneity and apparent discrepancies observed across early studies [12,20,21,22]. Indeed, only a minor fraction of PBMC appears to harbor replicative virus, and negative-strand RNA is typically present at levels close to the limits of detection, thereby reducing assay sensitivity and reproducibility. However, the progressive refinement of strand-specific molecular techniques, including the use of tagged primers, thermostable polymerases, and real-time PCR-based approaches, has substantially improved both the specificity and reliability of these assays, enabling a more consistent demonstration of HCV replication within lymphoid cells [20,21,23,24,25,26].

Thus, from a methodological perspective, detection of HCV negative-strand RNA, the low-abundance replicative intermediate rather than an mRNA, requires substantially greater stringency than detection of genomic positive-strand RNA. Early conventional strand-specific RT-PCR assays were vulnerable to nonspecific amplification of the abundant positive strand, and some protocols initially described as strand-specific were subsequently shown to provide incomplete strand discrimination. Accordingly, negative-strand positivity should be considered persuasive only when strand specificity has been experimentally validated. The most reliable and recent approaches combine high-temperature, strand-specific reverse transcription using thermostable *Thermus thermophilus* polymerase, synthetic positive- and negative-strand RNA standards tested across a wide concentration range, mock-extraction and no-template controls, and confirmation of the amplification product by nucleic-acid hybridization, melting-curve analysis, or sequencing. In the study by Skardasi et al., the strand-specific RT-PCR assay detected approximately 10^2^ copies of authentic negative-strand RNA per reaction, whereas nonspecific detection of the positive strand occurred only at concentrations of approximately 10^6^ copies or higher; the molecular findings were further supported by intracellular NS5A/core proteins, inhibition by the HCV-specific protease inhibitor telaprevir, the emergence of cell-associated viral variants, and the production of HCV RNA-containing particles [27]. Wróblewska et al. similarly employed high-temperature Tth-mediated reverse transcription followed by real-time PCR, parallel positive and negative controls, and product confirmation by melting-curve analysis and sequencing [28], whereas Pawełczyk et al. increased biological specificity by analysing purified PBMC subsets and combining negative-strand detection with intracellular NS3 protein assessment [29]. Mitogen stimulation can increase assay sensitivity, as reported by Pham and Michalak and used in some PBMC studies [18,30,31]; however, a positive result obtained after ex vivo stimulation should be interpreted as evidence of inducible replication under culture conditions rather than as a direct measurement of replication in freshly isolated circulating cells. Mechanistic studies identifying B7.2/CD86 as a co-receptor for lymphotropic HCV and demonstrating strain-dependent infection of memory B cells further support the biological plausibility of lymphoid infection, but do not establish its frequency in vivo [32]. Consistent with this caution, recent clinical studies still have produced heterogeneous results: Devi et al. detected low-level total HCV RNA in PBMCs but no negative strand in any of 12 patients [33], whereas the long-term study by Wróblewska et al. associated previously determined PBMC HCV-RNA status with adverse outcomes without introducing a new analytical validation of the assay [34]. Similar results of this latter study were reported by another recent paper confirming the persistence of HCV RNA in PBMCs of patients with DAA-induced SVR [35].

Although the identification of replicative intermediates in PBMC remains technically demanding, accumulating evidence seems to support the presence of low-level but genuine HCV replication within immune cells [11,12,18,20,21,24].

Thus, low-level HCV replication in lymphoid cells is biologically plausible and can be demonstrated under rigorously controlled experimental conditions, but it is not uniformly detected, and direct lymphoid infection should therefore be presented as one possible initiating mechanism, alongside chronic antigenic stimulation and bystander immune activation, rather than as a necessary or continuous driver of HCV-associated lymphomagenesis.

Importantly, viral infection is not uniformly distributed across PBMC subsets, and several studies indicate a preferential tropism for B lymphocytes, in which both viral genomes and replicative intermediates are more frequently detected [15,36,37,38,39]. Nevertheless, HCV RNA and, in some cases, markers of replication have also been identified in other leukocyte populations, including T lymphocytes, monocytes/macrophages, dendritic cells, and hematopoietic progenitor cells under specific conditions [27,40,41,42,43,44].

Both viral and host-related factors appear to modulate the extent of lymphotropism, including viral genotype, strain-specific characteristics, and the immunological status of the host [8,45,46]. In this context, molecular analyses have consistently demonstrated the existence of compartmentalized viral quasispecies among liver, plasma, and circulating immune cells, as well as across distinct leukocyte subsets [36,47,48]. This compartmentalization suggests that selective replication and local evolutionary pressures may occur within lymphoid microenvironments, thereby supporting the concept of partially independent viral dynamics in extrahepatic sites [36,47].

From a pathogenetic perspective, infection of lymphoid cells may contribute significantly to viral persistence by establishing an extrahepatic reservoir capable of sustaining chronic infection, particularly within long-lived or proliferating hematopoietic populations [12,14,17,43]. The prevalence of PBMC infection appears to increase with the duration of disease and has been variably associated with treatment response, relapse following antiviral therapy, and post-transplant recurrence [12,25,49].

Notably, the preferential interaction of HCV with B lymphocytes provides a biologically plausible link between chronic infection and lymphoproliferative disorders. Persistent antigenic stimulation, together with dysregulation of apoptotic pathways and the increased frequency of genetic alterations, most notably the first identified was the translocation (14;18)—t(14;18)—that leading to Bcl-2 overexpression, may promote prolonged B-cell survival and clonal expansion [50,51,52,53]. These mechanisms are thought to underlie the development of HCV-associated extrahepatic manifestations, including MC and, in a subset of patients, B-cell non-Hodgkin lymphoma [51,53,54,55,56].

Collectively, current evidence supports HCV lymphotropism as a highly plausible, and context-dependent contributor to HCV-associated lymphoproliferation, although the pathogenetic association can also be explained by indirect chronic antigenic and inflammatory mechanisms. Importantly, the persistence of clonally expanded B-cell populations after successful HCV eradication is compatible with an initiating role for HCV followed by virus-independent clonal maintenance.

**Figure 1:**
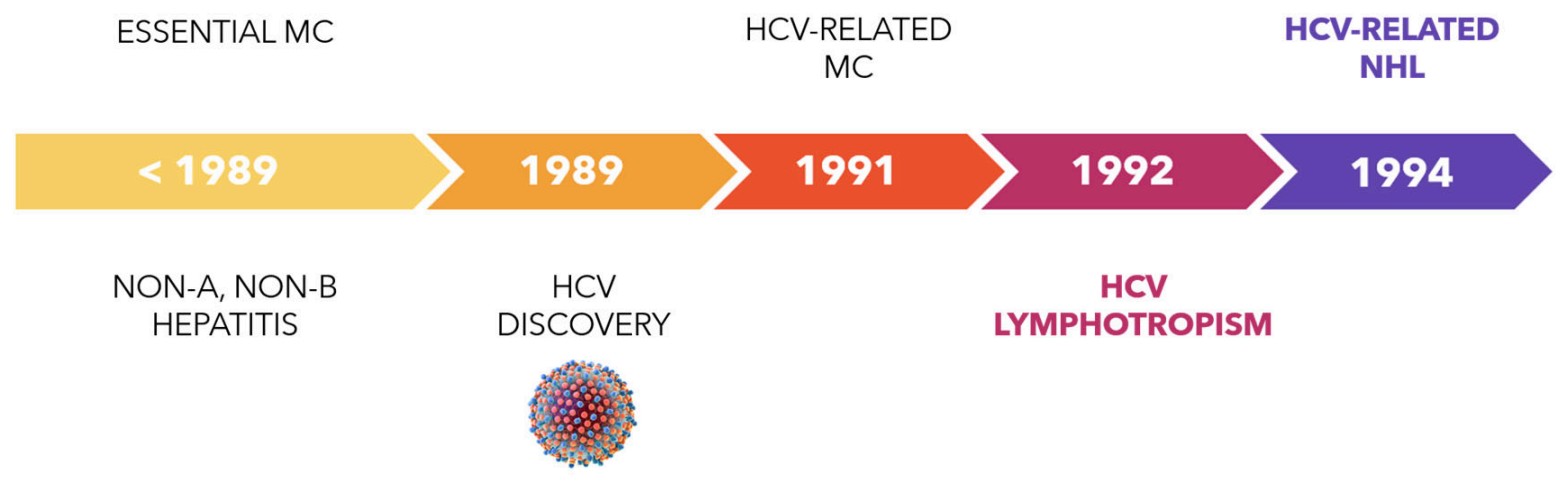
Historical milestones in the recognition of HCV-related lymphoma. Before the discovery of hepatitis C virus, most cases of the prelymphomatous mixed cryoglobulinemia were classified as “essential”. The identification of HCV in 1989 was followed by the demonstration, in 1991, of a strong association between active HCV infection and mixed cryoglobulinemia. In 1992, evidence of HCV lymphotropism and infection of peripheral blood mononuclear cells provided further insight into the immune and lymphoproliferative manifestations of the disease. By 1994, HCV infection had been associated with B-cell non-Hodgkin lymphoma, supporting a pathogenic continuum from chronic viral infection to mixed cryoglobulinemia and overt lymphoid malignancy. MC: Mixed Cryoglobulinemia; NHL: non-Hodgkin lymphoma.

The historical progression from the initial recognition of HCV infection to the identification of its association with mixed cryoglobulinemia first and then B-NHL is summarized in Figure 1.

## 4. Cryoglobulinemia as a Pre-Lymphomatous Condition

Chronic HCV infection is one of the most established infectious triggers of B-cell lymphoproliferative disorders. Epidemiological studies have consistently demonstrated a significantly increased risk of NHL in HCV-infected individuals, with relative risks ranging from approximately two- to three-fold compared with the general population [57,58,59]. Among the extrahepatic manifestations of HCV infection, type II MC represents the prototypical HCV-driven lymphoproliferative disorder and provides a unique model for studying the earliest stages of lymphomagenesis. However, MC should be regarded as a risk state and not as an obligatory intermediate in a deterministic HCV-to-lymphoma sequence.

In MC, chronic antigenic stimulation promotes the expansion of autoreactive B-cell clones producing immunoglobulins with RF activity. Immunogenetic analyses have demonstrated that these clones frequently exhibit restricted immunoglobulin gene usage, most commonly involving *IGHV1-69* paired with κ light chains such as *IGKV3-20* or *IGKV3-15*, consistent with antigen-driven clonal selection [60,61].

Importantly, selecting antigen is not necessarily viral in every patient: stereotyped B-cell receptors may recognize viral antigens, HCV-containing immune complexes, or self-IgG. Thus, restricted BCR repertoires support antigen-driven selection but do not establish universal HCV-specific antigen recognition.

Recent BCR-repertoire analyses further support this model of HCV as the initial trigger of a process that later becomes virus-independent. In patients with chronic HCV infection, including individuals who had achieved sustained virological response (SVR) after DAA therapy, B-cell repertoires showed increased richness, high levels of somatic hypermutation, skewing toward *IGHV1-69* and *IGHV4-59* rearrangements, and highly connected stereotyped CDR3 networks. Notably, these repertoire abnormalities persisted after HCV eradication, suggesting that HCV can leave a long-lasting imprint on the B-cell compartment rather than inducing only a transient antigen-driven response [62].

Importantly, the same immunoglobulin rearrangements detected in cryoglobulinemia have also been identified in HCV-associated lymphomas, suggesting that malignant clones may arise from previously expanded autoreactive B cells involved in cryoglobulin production. In line with this concept, *IGHV1-69* mutations associated with highly neutralizing anti-HCV antibodies have been shown to overlap with recurrent *IGHV1-69* point mutations found in clonotypic BCRs of high-grade B-cell lymphomas. This observation suggests that the anti-HCV humoral response, while potentially protective, may also select B-cell clones with lymphoma-like features [62]. Nevertheless, as the authors stated: the “*sequence-based approach is not definitive proof of the HCV-neutralizing capacity of the mutated BCRs and may be interpreted as a study limitation*” and “*proposed model of HCV-induced immune imprinting does not necessarily mandate that all IGHV1-69 BCRs exhibit a high neutralizing capacity*” [62]. Schultheiß and colleagues, rather, suggested that “*the multistep affinity maturation process triggered by an anti-HCV immune response may generate increased numbers of IGHV1-69–encoded BCRs with the described point mutations*” [62]. Thus, such persistence is compatible with a durable imprint of the original viral and inflammatory environment, but it may also reflect continued selection by self-antigens or progressive molecular autonomy of previously expanded clones rather than ongoing dependence on viral antigen.

Long-term follow-up studies indicate that approximately 5–10% of patients with MC eventually develop overt B-cell lymphoma, supporting the concept that MC represents a pre-lymphomatous risk state within the spectrum of HCV-associated lymphoproliferative disorders [63,64].

At the cellular level, these clonally expanded B cells predominantly accumulate within the CD21^low^ memory B-cell compartment, a subset characterized by autoreactivity, immune exhaustion, and impaired BCR signalling. Functional studies have shown that these cells exhibit defective calcium mobilization following BCR engagement and reduced proliferative responses to BCR or Toll-like receptor stimulation, indicating the presence of regulatory mechanisms that restrain uncontrolled activation [65,66].

This functional restraint suggests that early clonal expansion in cryoglobulinemia occurs within a context of immune tolerance rather than overt malignancy. The transition from this state to lymphoma therefore likely requires additional genetic and epigenetic events capable of overcoming these regulatory constraints.

Within this framework, persistent antigenic stimulation emerges as a major initiating and selective pressure rather than a continuous driver required at every stage of lymphomagenesis. Engagement of B-cell receptors by viral antigens, HCV-containing immune complexes, or self-antigens can sustain early B-cell activation and proliferation, thereby increasing the opportunity for clonal selection and acquisition of genetic alterations over time [60,67]. This chronic stimulation not only supports clonal expansion but also provides the substrate for early steps of clonal evolution: once relevant molecular lesions have accumulated; however, continued dependence on the original viral stimulus may progressively diminish.

Several complementary mechanisms further amplify this initially antigen-triggered process. Viral proteins can directly modulate B-cell signaling pathways; in particular, the HCV envelope glycoprotein E2 has been shown to interact with the CD81 receptor on B cells, triggering downstream signaling cascades that promote activation, proliferation, and survival [68].

These observations support at least two non-mutually exclusive routes of early B-cell selection: a viral-antigen-dominant route, in which HCV antigens or HCV-containing immune complexes contribute directly to BCR selection, and an autoantigen/bystander-dominant route, in which HCV acts primarily as an initiating inflammatory trigger and autoreactive clones are subsequently selected by self-antigens or sustained immune dysregulation. In both scenarios, later acquisition of genetic and epigenetic alterations can allow clonal persistence after viral eradication. Future studies that directly define BCR antigen specificity and integrate it with longitudinal clonal genomic profiling will be important to determine whether these routes confer different risks of lymphomatous progression and require different surveillance strategies.

## 5. BAFF Signaling as a Central Driver of B-Cell Survival and Clonal Persistence

Beyond antigenic drive, increasing evidence indicates that, in HCV-related lymphoproliferative disorders (LPDs), a complex cytokine network critically shapes the microenvironment that supports B-cell survival and clonal expansion [69]. Among these factors, B-cell activating factor (BAFF, also known as TNFSF13B) has emerged as a central regulator of B-cell homeostasis, differentiation, and peripheral tolerance. BAFF exerts its biological effects through three receptors -BAFF-R (BR3), transmembrane activator and CAML interactor (TACI), and B-cell maturation antigen (BCMA)- which activate canonical and non-canonical NF-κB, PI3K/AKT, and MAPK signaling pathways, thereby promoting B-cell survival and expansion [70].

While physiologically required for B-cell maturation and immune competence, sustained BAFF signaling can permit the survival of autoreactive B-cell clones that would otherwise be eliminated during peripheral tolerance checkpoints, thus creating a permissive environment for lymphoproliferation [71,72,73,74]. In the setting of chronic HCV infection, dysregulated BAFF expression has been consistently associated with the expansion and persistence of autoreactive B-cell clones characteristic of MC and related lymphoproliferative disorders [67,71,72,73,74].

Genetic predisposition further modulates this axis. Polymorphisms in both the BAFF promoter and BAFF receptor genes are enriched in patients with HCV-associated cryoglobulinemic vasculitis and non-Hodgkin lymphoma, indicating that host genetic variation influences susceptibility to virus-driven B-cell expansion [75,76,77,78]. These findings highlight the interplay between chronic infection, cytokine signaling, and host genetic background in determining disease evolution.

Importantly, BAFF does not act in isolation but rather within a broader cytokine network that contributes to B-cell activation and survival. Elevated levels of interleukin-6 (IL-6), interleukin-10 (IL-10), and other pro-survival cytokines have been described in HCV-associated lymphoproliferative disorders, where they promote plasma cell differentiation, immune dysregulation, and resistance to apoptosis [67,69]. These cytokines cooperate to establish a chronically activated yet immunoregulatory microenvironment that sustains long-lived autoreactive B-cell populations.

Within this context, BAFF signaling emerges as a central hub integrating antigenic stimulation and cytokine-mediated survival cues. Beyond its role in early B-cell activation, BAFF contributes to the long-term persistence of pathogenic clones and may also support the survival of transformed cells. Indeed, BAFF-mediated signaling has been shown to synergize with anti-apoptotic pathways, particularly BCL-2-dependent mechanisms, thereby enhancing resistance to apoptosis [79].

The central role of the BAFF axis is further underscored by emerging therapeutic strategies targeting this pathway. Preclinical studies have demonstrated that a humanized monoclonal antibody directed against BAFF-R exerts broad cytotoxic activity against B-cell malignancies [80]. Similarly, BAFF-R-targeted CAR-T cell approaches have shown promising activity in selectively eliminating BAFF-R-positive malignant B cells [81]. Nevertheless, despite their potential interest in HCV-related lymphoproliferative disorders, BAFF-R-directed antibodies and CAR-T cells have not been specifically investigated in HCV-associated NHL or cryoglobulinemic vasculitis. Thus, their possible therapeutic relevance in these settings remains, at present, largely theoretical and hypothesis-generating, and there is currently no direct clinical evidence demonstrating their efficacy in HCV-related CV or lymphoma.

Taken together, these observations support a model in which chronic antigenic stimulation and cytokine-mediated signaling, centered on BAFF but reinforced by additional cytokines such as IL-6 and IL-10, cooperate to sustain the long-term persistence of autoreactive B-cell clones. This cytokine-driven survival advantage prolongs the lifespan of antigen-selected B-cell populations and increases their cumulative exposure to mutagenic processes associated with chronic immune activation. Over time, this permissive microenvironment facilitates the gradual accumulation of somatic genetic alterations, thereby promoting progression along the spectrum from benign antigen-driven lymphoproliferation to overt malignancy.

In this framework, BAFF signaling represents a key component of the early immunological landscape that sustains clonal survival and evolution, whereas the transition toward lymphoma ultimately depends on the acquisition of oncogenic genetic lesions affecting critical regulatory pathways. Accordingly, the next step in the molecular evolution of HCV-associated lymphoproliferative disorders involves the progressive accumulation of genetic alterations within these persistent B-cell clones, driven by genomic instability and dysregulated mechanisms of somatic hypermutation.

## 6. Genetic Instability and Genomic Evolution Toward Overt Lymphoma

The transition from reversible, antigen-dependent B-cell expansion to autonomous lymphomagenesis requires the progressive acquisition and selection of genetic lesions. Within the permissive microenvironment created by chronic antigenic stimulation and BAFF-mediated survival signalling, repeatedly activated B-cell clones undergo prolonged proliferation and selection, increasing the opportunity for DNA damage, somatic evolution and the emergence of increasingly autonomous subclones.

A central contributor to this process is activation-induced cytidine deaminase (AID), the enzyme responsible for initiating somatic hypermutation and immunoglobulin class-switch recombination [82]. Under physiological conditions, AID activity is tightly regulated and largely restricted to activated germinal-centre B cells. However, prolonged or ectopic AID expression can generate off-target mutations and chromosomal rearrangements involving non-immunoglobulin genes, thereby contributing to lymphomagenesis [83,84]. The physiological role of AID in antibody diversification and its oncogenic potential have been extensively reviewed [85].

Experimental studies have suggested that HCV may reinforce these mutagenic processes. Machida and colleagues reported that HCV exposure induced a mutator phenotype associated with increased expression of AID and error-prone DNA polymerases, resulting in enhanced mutation frequencies at both immunoglobulin loci and proto-oncogenes [86]. Because these findings were obtained primarily in experimental models, they should be interpreted as mechanistic support for HCV-associated mutagenesis rather than definitive evidence that direct infection of B cells represents the predominant in vivo mechanism.

Recent genomic analyses have provided further insight into the early genetic events associated with HCV-related cryoglobulinemia. Deep sequencing approaches revealed that pathogenic IgM-expressing B-cell clones may harbor a high somatic mutational burden together with recurrent lymphoma-associated driver alterations. In particular, the genomic study by Young and colleagues demonstrated that expanded autoreactive B-cell clones can accumulate multiple driver mutations affecting genes such as *NOTCH1*, *KLF2*, *TRAF3*, and *BIRC3*, as well as cytogenetic abnormalities including trisomy 12 [87].

Phylogenetic reconstruction indicated that these driver alterations represented truncal events that preceded subsequent intraclonal diversification of immunoglobulin genes and the acquisition of stronger rheumatoid-factor activity. Thus, at least in the investigated cases, lymphoma-associated mutations arose relatively early during clonal evolution, before complete antibody affinity maturation. The pathogenic clones recognized self-IgG rather than HCV E2, indicating that molecular mimicry between viral and self-antigens is not required in every case of HCV-associated cryoglobulinaemia. In other words, this raises the possibility that, once immune dysregulation has been initiated, autoantigen-driven selection may be sufficient to maintain or further shape pathogenic clones. It also supports pathogenetic heterogeneity among patients: some clones may remain predominantly linked to viral antigens or virus-containing immune complexes, whereas others may be sustained by self-antigen recognition and acquired molecular lesions.

However, because the study included only a limited number of patients, these observations require confirmation in larger and clinically heterogeneous cohorts [54].

Many of these early alterations converge on pathways controlling B-cell activation, survival and proliferation. Loss-of-function lesions affecting *KLF2*, *TRAF3* or *BIRC3* may remove negative constraints on NF-κB signalling and increase responsiveness to BAFF (see above), CD40 and Toll-like receptor stimulation. Conversely, activating NOTCH1 alterations, previously reported by other authors [88], and trisomy 12 may provide additional proliferative and survival advantages.

Importantly, genetically evolved B-cell clones may persist after successful HCV eradication [52,89,90].

These observations further support an explicit two-phase conceptual model. During an initiation phase, HCV provides a selective and inflammatory environment through chronic antigenic stimulation, immune-complex/BCR engagement, cytokine-mediated survival signals, and possibly direct lymphoid infection. During a subsequent progression phase, acquired driver mutations, epigenetic remodeling, altered survival pathways, and, in some cases, persistent autoantigen-driven stimulation can render selected clones partially or fully independent of the original viral stimulus. Removal of HCV therefore reduces ongoing immune activation but may not eliminate clones that have already acquired molecular autonomy, confirming HCV as an important initiating trigger and early selective pressure, whereas continued viral presence is not required for malignant clonal maintenance in all patients.

In other words, as clonal autonomy increases, this early genetic evolution may culminate in overt B-cell lymphoma. DLBCL represents the predominant aggressive lymphoma subtype reported in association with chronic HCV infection and may develop either de novo or through transformation of a pre-existing indolent lymphoma, particularly MZL [88,91].

In fact, among HCV-associated lymphomas, Splenic Marginal Zone Lymphomas (SMZL) provides particularly compelling clinical evidence that HCV can act as an initiating and, in selected cases, sustaining lymphomagenic stimulus. In the historical series of HCV-positive splenic lymphoma with villous lymphocytes, an entity within the SMZL spectrum, all nine patients showed hematological regression only after HCV-RNA clearance (eight complete and one partial remission) and one patient relapsed when viremia recurred [92]. In the expanded 18-patient series, 14 patients (78%) achieved a sustained complete hematological response after viral clearance, although monoclonal immunoglobulin gene rearrangements persisted [93]. In the DAA era, the prospective FIL_BArT study enrolled 40 untreated patients with HCV-positive indolent lymphoma, including 27 with MZL and six with SMZL: all achieved SVR, while 45% achieved an objective lymphoma response, including 20% complete responses [94]. Although the trial was not powered for subtype-specific comparisons, these observations show that removal of the viral trigger alone can induce tumor regression in a substantial subset of indolent lymphomas, strongly supporting an HCV-dependent process (most plausibly mediated by chronic antigenic and inflammatory stimulation), while not excluding a direct lymphotropic contribution. Conversely, incomplete responses and persistent molecular clonality after SVR indicate that some clones have already acquired partial autonomy [1,89]. This biological evolution observed in some cases is mirrored by the markedly asymmetric molecular architecture of HCV-positive DLBCL. In the series by Sciarra et al., rearrangements involved *BCL6* in 50.9% and *MYC* in 11.3% of cases, but *BCL2* in only 3.7%; earlier case–control data similarly identified *BCL2* rearrangements in 0% of HCV-positive de novo DLBCLs versus 19% of HCV-negative controls [91,95]. Recurrent mutations affected *KMT2D* (42.6%), *SETD1B* (33.3%), *RERE* (29.4%), *FAS* and *PIM1* (27.8% each), and *TBL1XR1* (25.9%), whereas NOTCH-pathway lesions (particularly involving *NOTCH2* and less frequently *NOTCH1* or *SPEN*) occurred in approximately one quarter of cases [88,91]. Together with an underlying MZL component in 14.8% of tumors and assignment to the BN2 subtype in 48.2% of classifiable cases, this profile closely resembles marginal-zone differentiation and supports transformation of an HCV-selected MZL-like clone in a substantial subgroup, without implying that all HCV-associated DLBCLs arise through this route [88,91].

In line with these latter observations, genomic studies of overt HCV-associated lymphomas/lymphoproliferations have revealed a heterogeneous spectrum of somatic alterations involving B-cell receptor–NF-κB signalling, NOTCH signalling, chromatin regulation, apoptotic control, immune escape and cell-cycle progression [87,91,92].

A whole-exome sequencing study of HCV-associated B-cell lymphomas identified alterations affecting B-cell receptor–NF-κB signalling in approximately 62% of cases, followed by abnormalities involving epigenetic and chromatin regulators, the NOTCH pathway, MAPK–ERK signalling, JAK–STAT signalling, immune escape and cell-cycle regulation [92]. Recurrent copy-number abnormalities included trisomy 3 or 3q gain, 6q deletion, 8q gain, trisomy 18 and trisomy 12. Genes such as *NOTCH2*, *DTX1*, *KMT2D* and *ZFP36L1* emerged as potentially relevant, although the overall findings did not identify a single molecular alteration uniquely attributable to HCV infection.

More detailed genomic characterization of HCV-associated DLBCL has confirmed the importance of epigenetic and transcriptional regulators. Sciarra and colleagues identified recurrent mutations in *NOTCH*, *KMT2D*, *SETD1B*, *RERE*, *FAS*, *PIM1* and *TBL1XR1*. BCL6 rearrangements were relatively frequent, whereas BCL2 rearrangements were uncommon [91,93]. These observations are consistent with the broader molecular heterogeneity of DLBCL but suggest that HCV-associated cases may be enriched for selected genomic configurations rather than constituting a completely distinct molecular entity.

Alterations of the NOTCH pathway represent one of the most reproducible features of HCV-associated DLBCL. Arcaini and colleagues first demonstrated that these lymphomas frequently harbour NOTCH-pathway mutations, predominantly involving *NOTCH2*, together with less frequent *NOTCH1* and *SPEN* alterations [88]. These abnormalities were enriched in HCV-positive compared with HCV-negative DLBCL and were associated with residual low-grade or marginal-zone-like lymphoma components, supporting the hypothesis that a subset of HCV-associated DLBCLs develops through transformation of an underlying MZL clone [88].

Consistent with these findings, Sciarra and colleagues detected NOTCH-pathway mutations in approximately one quarter of HCV-associated DLBCLs and identified an accompanying MZL component in a subset of cases [91]. Among lymphomas that could be assigned to a LymphGen molecular subtype, the BN2 subtype, characterized by frequent *NOTCH2* and *BCL6* alterations and biologically related to marginal-zone differentiation, was the most common [91]. NOTCH-pathway alterations were also associated with increased lymphoma-related mortality. Collectively, as already mentioned, these observations support a marginal-zone origin for a substantial proportion of HCV-associated DLBCLs, although they do not imply that all cases arise through histological transformation.

Host genetic susceptibility should nevertheless be distinguished from the somatic alterations acquired by the expanding B-cell clone. A genome-wide association study of HCV-associated cryoglobulinaemic vasculitis identified a susceptibility locus within the extended major histocompatibility complex on chromosome 6, close to the *NOTCH4* and class II *HLA* regions [94]. Subsequent analyses of patients across the spectrum of HCV-related benign and malignant lymphoproliferative disorders further supported the involvement of *NOTCH4* and MHC class II variants in disease susceptibility and progression [95,96].

More recently, Fu and colleagues used Mendelian randomization and colocalization analyses to investigate whether the association between chronic HCV infection and DLBCL might be causal rather than purely observational [97]. Genetically predicted chronic hepatitis C was associated with an increased risk of DLBCL, whereas comparable associations were not observed for all B-cell lymphoma subtypes examined. Two colocalized variants, *rs17208853* and *rs482759*, were mapped to a chromosome 6 region containing *BTNL2*, *NOTCH4* and the long non-coding RNA *TSBP1-AS1*. These findings are conceptually consistent with previous studies implicating the extended *MHC–NOTCH4* region in HCV-associated LPDs [94,96]. However, because the analysis was based on a limited number of genetic instruments and predominantly European datasets, it provides supportive genetic-epidemiological evidence rather than a direct demonstration of the cellular mechanisms underlying malignant transformation.

Taken together, the available evidence is consistent with a multistep model of HCV-associated lymphomagenesis, but it does not prospectively validate a single obligatory HCV→MC→MZL→DLBCL sequence. Chronic antigenic and inflammatory stimulation and cytokine-mediated survival signals may initially favour the expansion and persistence of autoreactive B-cell clones. Continued proliferation, apoptosis escape [50], AID-associated mutagenesis [86], age-related somatic evolution, and host susceptibility can then promote the acquisition of driver lesions affecting *NF-κB*, *NOTCH*, and related pathways [87,88,91,92,93]. Additional genetic, epigenetic, cytogenetic, and microenvironmental events may progressively increase clonal autonomy and lead to lymphoma. In some patients, an indolent marginal-zone-like clone may transform to DLBCL, whereas other HCV-associated aggressive lymphomas may arise through alternative pathways without a documented MC or MZL intermediate [91,92,98]. The fact that only a minority of patients with MC develop lymphoma indicates that critical rate-limiting cofactors remain unidentified. Thus, the proposed model should be viewed as a conceptual framework encompassing multiple possible routes rather than a deterministic continuum. Large prospective longitudinal cohorts comparing progressors and non-progressors are needed to define the relevant cofactors, temporal sequence of molecular events, and validated predictive biomarkers.

**Figure 2:**
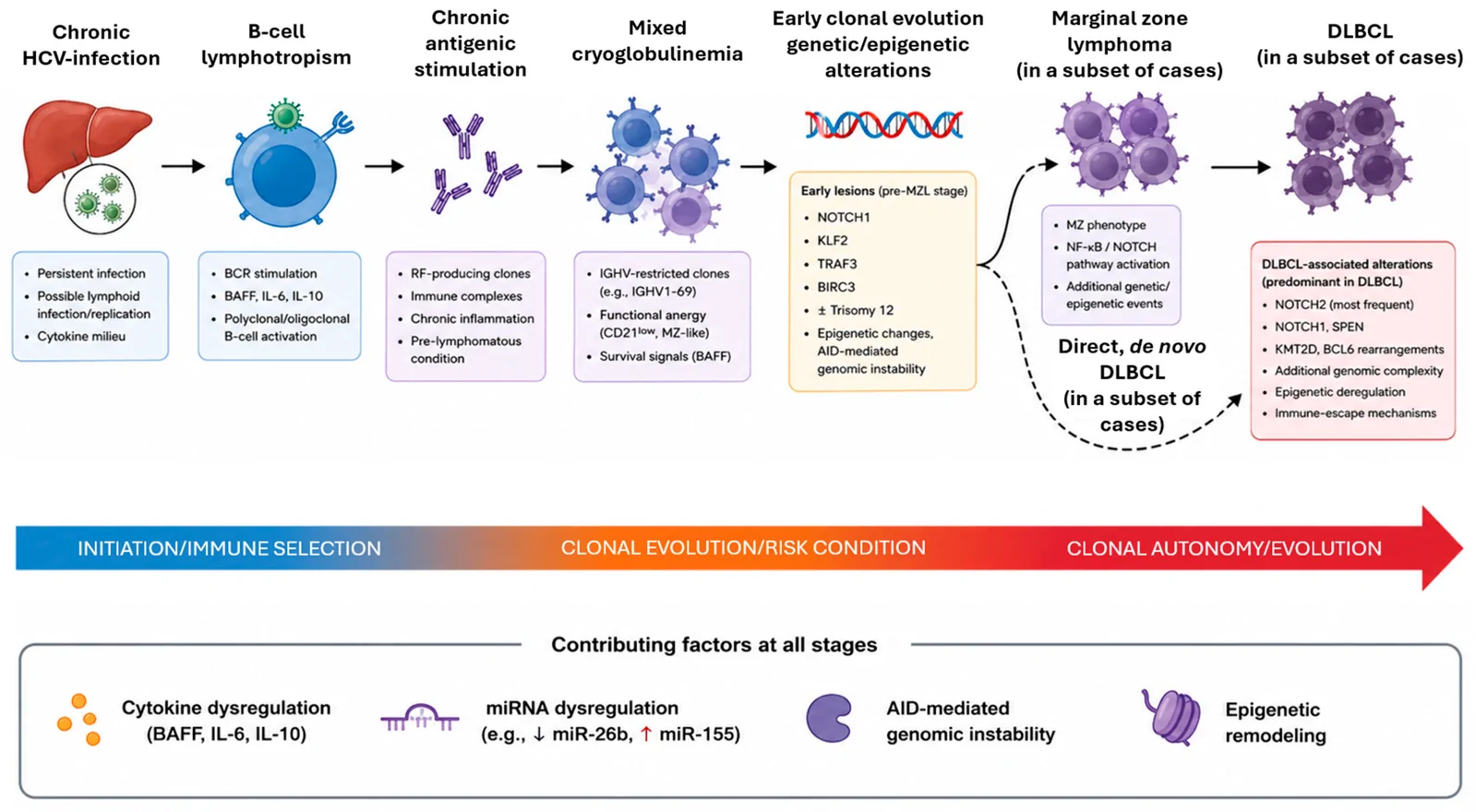
Conceptual multistep model of HCV-driven B-cell lymphomagenesis.

A simplified version of the proposed multistep model linking chronic HCV infection to B-cell clonal expansion, marginal zone lymphoma, and eventual/direct transformation to DLBCL is summarized in Figure 2.

During an initiation phase, chronic HCV infection may provide antigenic, inflammatory, cytokine-mediated, and possibly direct lymphoid stimuli that promote autoreactive B-cell expansion and, in some patients, mixed cryoglobulinemia. During a progression phase, additional genetic, epigenetic, cytogenetic, and microenvironmental ‘hits’ may confer increasing clonal autonomy and permit development of MZL or DLBCL, either through histological transformation or through direct alternative routes. Because only a part of patients with mixed cryoglobulinemia progress to lymphoma, cofactors and validated, predictive biomarkers remain to be defined. The model was partially created with AI-assisted illustration tools.

## 7. Transcriptomic Remodelling and Epigenetics in HCV-Associated Lymphoproliferation

Beyond the accumulation of genetic lesions, increasing evidence indicates that epigenetic and transcriptional reprogramming represent critical events in the transition from antigen-driven lymphoproliferation to overt lymphoma. Transcriptomic profiling studies of peripheral B cells from patients with HCV-associated lymphoproliferative disorders have revealed a distinctive transcriptional landscape characterized by the coexistence of clonal expansion and persistent immune inhibitory signaling [65]. RNA-sequencing analyses demonstrated that peripheral B cells from patients with HCV-associated B-cell lymphoma display an anergic-like transcriptional signature, including enrichment of genes involved in inhibitory receptor signaling, immune tolerance, and repression of B-cell receptor signaling pathways, together with expansion of autoreactive clonotypes associated with autoimmunity and lymphoproliferation [99]. This confirms previous studies of Visentini M. and colleagues showing that the dominant IGHV1-69^+^ IgM^+^CD27^+^ B-cell clones in HCV-related MC include both marginal-zone-like CD21^high^ cells and atypical CD21^low^CD11^+^ cells; however, both subsets display functional anergy/exhaustion, failing to proliferate or differentiate into plasmablasts following BCR or TLR7/9 stimulation and showing increased expression of the antiproliferative transcriptional regulator Stra13 [66,100]. HCV eradication generally reduces the size of these clones and reverses some anergy-associated signals, although TLR9-unresponsive marginal-zone-like cells may persist for months or years after viral clearance [101,102]. The same Italian authors further proposed that progression toward splenic marginal zone lymphoma may involve disruption of this protective restraint, with Stra13 down-regulation and partial recovery of TLR9-driven proliferation, thereby allowing chronically stimulated autoreactive clones to acquire greater proliferative autonomy [103].

Importantly, the transcriptional programs emerging from the analysis of Henning and coworkers coexist with increased expression of genes involved in chromatin remodeling, histone modification, and epigenetic regulation, suggesting that epigenetic remodeling may enable previously anergic B-cell populations to bypass proliferative restraints while maintaining molecular features of their antigen-driven origin [99]. This concept is supported by the previously described deep genomic analyses of HCV-related cryoglobulinemic vasculitis, which revealed that pathogenic IgM^+^ B-cell clones frequently harbor thousands of somatic mutations together with recurrent lymphoma-associated driver alterations, including mutations in *NOTCH1*, *KLF2*, *TRAF3*, and *BIRC3* or chromosomal abnormalities such as trisomy 12 [87]. Notably, these lesions appear to arise early in clonal evolution and likely confer selective survival advantages through dysregulation of NF-κB signaling pathways, thereby facilitating the expansion of autoreactive B-cell clones under conditions of chronic viral stimulation [87].

Further evidence for the stability of these pathogenic clones comes from longitudinal molecular studies performed after antiviral therapy. In patients with HCV-associated CV treated with DAAs, virological clearance and clinical remission do not necessarily lead to eradication of monoclonal B-cell populations. Detailed immunogenetic analyses demonstrated persistence of identical IGHV rearrangements and ongoing intraclonal diversification, and in some cases retention of lymphoma-associated genomic abnormalities such as *BCL2–IGH* rearrangements, indicating durable antigen imprinting and partial independence from viral stimulation [89].

Collectively, the genomic and transcriptomic observations support a model in which chronic HCV infection initially promotes expansion of autoreactive B-cell clones within an anergic functional framework. Subsequent epigenetic reprogramming and acquisition of driver mutations enable selected clones to escape regulatory constraints, persist even after viral eradication, and ultimately progress toward malignant transformation.

Among the epigenetic regulators, microRNAs (miRNAs) emerged as relevant players in human carcinogenesis, providing both minimally invasive biomarkers of disease evolution and functional regulators of the immune and oncogenic pathways that sustain lymphoproliferation. As stated above, in HCV infection, the best clinical model of a pre-lymphomatous condition is type II MC. In this context, miRNA profiling helps to conceptualize lymphomagenesis as a continuum in which chronic antigenic drive and inflammatory cues progressively reshape B-cell programs.

From a pathogenetic perspective, it is useful to first consider tissue-based miRNA alterations, which more directly reflect intratumoral regulatory programs, and then describe patterns of circulating miRNAs, as peripheral biomarkers reflecting functional cellular alterations (Supplementary Table S1).

### 7.1. miRNAs in Biopsies: Mechanistic Programs and HCV-Shaped Lymphoma Biology

A pivotal mechanistic insight comes from the seminal study by Peveling-Oberhag et al. [104], who analyzed SMZL and demonstrated that miR-26b is significantly downregulated specifically in HCV-positive SMZL, supporting a tumor-suppressive role for miR-26b and suggesting that HCV infection may imprint marginal zone lymphomagenesis through selective miRNA repression.

Another tissue profiling study indicated that HCV-associated lymphomas harbor etiology-specific miRNA signatures. In indolent B-cell NHL, Bruni et al. [105] analyzed formalin-fixed, paraffin-embedded (FFPE) lymph node tissues and demonstrated virus-associated differences in miRNA expression, notably miR-30b upregulation in HCV-positive cases, while miR-92a downregulation characterized virus-negative lymphomas, and subtype-restricted analyses (e.g., nodal marginal zone lymphoma) suggested associations for miR-223, miR-29a, and miR-29b with HCV infection. These findings underscored both the existence of HCV-imprinted molecular programs and the confounding role of histological heterogeneity in small cohorts [105].

In aggressive disease, Augello et al. [106] performed comprehensive miRNA profiling in HCV-associated DLBCL and identified a distinct 52-miRNA signature distinguishing tumor tissue from non-neoplastic germinal center B cells. Importantly, pathway analyses showed enrichment of networks involved in PI3K–Akt signaling, survival pathways, and viral carcinogenesis, and specific miRNAs (including reduced miR-138-5p and increased miR-147a/b and miR-511-5p) were associated with prognosis in HCV-positive DLBCL, indicating that miRNA programs may stratify clinical behavior within this etiologic subset of a high-grade lymphoma [106].

These observations have been further integrated with a dysregulation of the miR-17-92 cluster in HCV-related CV, coupled with increased c-Myc expression, suggesting engagement of canonical oncogenic circuits already at the pre-lymphomatous stage [107]. Collectively, tissue studies support the concept that chronic HCV infection shapes lymphoma biology through selective modulation of miRNAs involved in proliferation, survival, and B-cell differentiation.

### 7.2. miRNAs as Biomarkers in Peripheral Blood and Other Biofluids

Peripheral blood-based miRNA alterations can be exploited for risk stratification and longitudinal monitoring across the HCV, MC, lymphoma trajectory. In a large PBMC study comparing HCV patients without LPD, HCV-MC, and HCV-NHL, miR-26b emerged as a particularly informative marker: it was significantly downregulated in both MC and NHL, whereas miR-21/miR-16/miR-155 were increased only in NHL PBMCs, suggesting stage-specific miRNA signatures (pre-lymphomatous versus malignant) [108]. Importantly, miR-26b downregulation, previously suggested by Peveling-Oberhag and colleagues in HCV-related lymphoma tissues [104], was reversible: it normalized after antiviral therapy in MC patients achieving complete virological and clinical response, supporting its utility as a dynamic biomarker linked to disease activity and possibly to progression risk [108].

Complementary PBMC data further support this concept of peripheral miRNA profiling to detect and even help discriminate HCV-related malignancies, with miR-155/miR-16 alterations appearing more lymphoma-restricted [109]. Beyond PBMCs, biofluid-based signals may also reflect intercellular communication: serum-derived and hepatocyte-derived exosomes carrying miRNAs (notably exo-miR-122, exo-let-7b, exo-miR-206) can induce TLR7/NF-κB activation in macrophage-lineage cells and increase BAFF, thereby generating a survival/activation milieu for B cells; in addition to mechanistic implications, this supports the rationale for exploring exosomal miRNAs as circulating readouts of HCV-driven immune activation relevant to B-cell expansion [110] (Supplementary Table S1).

To integrate these findings within a stage-specific framework, Table 1 summarizes the major mechanisms, molecular alterations, reversibility after DAA therapy, and candidate biomarkers across the progression from chronic HCV infection and MC/CV to MZL and DLBCL, highlighting the transition from predominantly antigen-dependent B-cell activation to increasing clonal autonomy.

Table 1 summarizes the major pathogenic mechanisms, representative molecular alterations, degree of reversibility after DAA therapy, and potential biomarkers across the proposed progression from chronic HCV infection MC/CV, MZL, and (DLBCL). Overall, early disease stages are predominantly characterized by antigen-dependent and microenvironment-supported B-cell activation, whereas the progressive acquisition of genetic, epigenetic, and transcriptional alterations is associated with increasing clonal autonomy and reduced dependence on the original viral stimulus. Accordingly, DAA-induced viral eradication may reverse immune activation and reduce clonal expansion in earlier stages, but established B-cell clones carrying driver lesions may persist after sustained virological response and overt lymphoma may require lymphoma-directed treatment. This classification is descriptive and does not represent a formal grade assessment. None of the proposed molecular or circulating biomarkers is currently validated to reliably predict progression from MC/CV to lymphoma at the individual-patient level.

## 8. Clinical Implications of Persistent B-Cell Clonality After SVR

The persistence of expanded B-cell clones after SVR has potentially important clinical implications. Although HCV eradication removes the initiating antigenic and inflammatory stimulus and is frequently associated with clinical improvement and reduction in B-cell expansion, identical IGHV clonotypes and, in some patients, lymphoma-associated molecular abnormalities may persist after DAA therapy [62,89,90]. Persistent clonality should not, however, be interpreted as synonymous with imminent malignant transformation, as its long-term prognostic significance and the absolute risk associated with individual molecular abnormalities remain not completely defined. Nevertheless, these findings support the potential value of post-DAA surveillance, particularly in patients with a previous HCV-associated lymphoproliferative disorder, persistent or recurrent cryoglobulinemic manifestations, or detectable clonal B-cell populations after SVR. Such follow-up may include periodic clinical and hematological assessment, with more detailed immunophenotypic or molecular investigations reserved for patients with persistent or evolving abnormalities [95]. Candidate biomarkers, including IGHV clonotypes, lymphoma-associated genomic lesions, BAFF-related signals, and circulating miRNA profiles, may ultimately contribute to risk stratification; however, their clinical utility remains investigational. Importantly, no validated criteria currently define which patients should undergo intensified surveillance, which biomarkers should be monitored, or the optimal frequency and duration of follow-up. Prospective longitudinal studies are therefore required to determine whether persistence or evolution of specific B-cell clones after SVR can reliably identify patients at increased risk of subsequent lymphoma and guide individualized surveillance strategies.

## 9. Conclusions

Current evidence supports a multistep model of HCV-associated lymphomagenesis. During an initiation phase, HCV may act primarily as a trigger and selective environment through chronic antigenic stimulation, immune-complex/B-cell receptor engagement, cytokine-mediated survival signals, and bystander immune activation; direct lymphoid infection may contribute in selected settings but remains technically difficult to establish. Mixed cryoglobulinemia represents a pre-lymphomatous risk state, as approximately 5–10% of affected patients progress to overt lymphoma, indicating that additional cofactors are necessary. During a progression phase, acquisition of somatic driver lesions, chromosomal abnormalities, transcriptomic changes, and epigenetic remodeling can allow selected clones to escape functional restraint and may become increasingly independent of the original viral stimulus. The observation that some pathogenic clones recognize self-IgG rather than HCV E2 further indicates that autoantigen-driven selection can contribute to clonal maintenance in at least a subset of patients. Lymphoma may therefore emerge through more than one route, including transformation of an indolent MZ-like clone or alternative pathways leading more directly to aggressive disease.

The persistence of large B-cell clones and lymphoma-associated molecular abnormalities after SVR demonstrates that successful viral eradication does not invariably eliminate clones that have already acquired molecular autonomy. This two-phase model has direct clinical implications: antiviral therapy remains essential for removing the initiating viral stimulus, but post-eradication surveillance is warranted in patients with persistent clonal populations or other high-risk features, and immunomodulatory or lymphoma-directed therapy may be required when clonal disease becomes virus-independent. Future prospective, multicenter longitudinal studies should compare progressors and non-progressors and integrate clinical features with BCR antigen specificity, immunoglobulin-repertoire analysis, germline susceptibility, somatic genomic alterations, transcriptomic and epigenetic profiles, and circulating biomarkers. Such studies are needed to identify the critical cofactors and molecular events, distinguish viral-antigen-dominant from autoantigen-dominant pathogenetic routes, establish progression timelines, and validate biomarkers for individualized surveillance and treatment.

More broadly, HCV-associated lymphomagenesis remains a paradigmatic model for understanding how chronic infection can initiate immune selection and clonal evolution that may subsequently become independent of the original infectious trigger, thereby contributing to human cancer development.

## Figures and Tables

**Table 1:** Stage-specific features of HCV-associated B-cell lymphomagenesis.

Disease Stage	Main Mechanisms	Key Molecular Alterations	Reversibility After DAAs	Potential Biomarkers	Ref.
Chronic HCV infection	Chronic antigenic stimulation; BCR activation; possible lymphoid infection; BAFF/cytokine-mediated survival	HCV RNA/replicative intermediates in PBMCs; t(14;18)/BCL2; skewed *IGHV1-69*/*IGHV4-59* repertoire; *BAFF*/*BAFF-R* and *MHC-NOTCH4* susceptibility variants	Mostly reversible at the antigenic/inflammatory level; selected clonotypes may persist	HCV RNA; BAFF; t(14;18); IGHV repertoire; exosomal miRNAs	[8,9,10,11,12,13,14,15,16,17,18,19,20,21,22,23,24,25,26,27,28,29,30,31,32,33,34,35,36,37,38,39,40,41,42,43,52,63,64,65,66,67,68,69,85,86,87,101]
MC/CV	Expansion of RF-producing autoreactive B cells; persistent antigen/self-antigen selection; functional anergy; cytokine-supported clonal survival	*IGHV1-69*, *IGKV3-20/3-15*; CD21low clones; *NOTCH1*, *KLF2*, *TRAF3*, *BIRC3*; trisomy 12; BCL2-IGH; miR-26b down; miR-17-92 dysregulation	Partial: clinical and clonal reduction common, but identical IGHV clones and genomic lesions may persist	Cryoglobulins/RF; complement; BAFF; IGHV clonotypes; BCL2-IGH; driver mutations; miR-26b	[40,41,42,43,44,45,46,47,48,49,50,51,52,53,54,55,56,62,63,64,65,66,67,68,69,78,80,85,86,87,91,92,93,94,95,96,97,98,99]
MZL	Escape from anergy; increasing clonal autonomy; NF-kB/NOTCH-mediated survival and proliferation	*KLF2*, *TRAF3*, *BIRC3*, *NOTCH* alterations; trisomy 12; epigenetic/transcriptomic remodeling; miR-26b down; miR-30b up	Variable; less reversible once driver alterations are established	Persistent IGHV clonotypes; NOTCH/NF-kB pathway lesions; miR-26b; miR-30b	[53,78,79,80,89,90,91,92,93,94,95,96]
DLBCL	Advanced clonal autonomy; genomic/epigenetic evolution; possible transformation from MZL or de novo development	*NOTCH2*/*NOTCH1*/*SPEN*, *KMT2D*, *SETD1B*, *FAS*, *PIM1*, *TBL1XR1*; *BCL6* rearrangements; CNAs; BN2 subtype; miR-138-5p down; miR-147a/b and miR-511-5p up	Limited: viral clearance alone is generally insufficient once overt lymphoma is established	NOTCH-pathway alterations; BCL6; molecular subtype; tissue/PBMC miRNA signatures	[79,82,83,84,85,86,87,88,89,90,97,99,100]

## Data Availability

No new data were created or analyzed in this study. Data sharing is not applicable.

## Supplementary Materials

The following supporting information can be downloaded at: https://www.mdpi.com/article/10.3390/cancers18172761/s1, Table S1. Key microRNAs implicated in HCV-associated B-cell lymphoproliferation/lymphomagenesis.

## Abbreviations

HCV	hepatitis C virus
MC	mixed cryoglobulinemia
IgG	immunoglobulin G
IgM	immunoglobulin M
RF	rheumatoid factor
CV	cryoglobulinemic vasculitis
NHL	non-Hodgkin lymphoma
DLBCL	diffuse large B-cell lymphoma
MZL	marginal zone lymphoma
DAA	direct-acting antiviral
PBMC	peripheral blood mononuclear cell
RNA	ribonucleic acid
PCR	polymerase chain reaction
BCR	B-cell receptor
CDR3	complementarity-determining region 3
CD	cluster of differentiation
BAFF	B-cell activating factor
LPD	lymphoproliferative disorder
BAFF-R	B-cell activating factor receptor
TACI	transmembrane activator and calcium-modulating cyclophilin ligand interactor
BCMA	B-cell maturation antigen
