# Supplementary material for: From Antigenic Drive to Clonal Autonomy: An Update on Molecular Mechanisms of HCV-Related B-Cell Lymphomagenesis
Source: Cancers (Basel). 2026 Aug 25;18(17):2761. doi: 10.3390/cancers18172761 (PMC13565320; doi:10.3390/cancers18172761)
Supplement: Supplementary file 1 [file cancers-18-02761-s001.zip › cancers-4485244-supplementary.pdf]

Supplementary Table 1. Key microRNAs implicated in HCV-associated B-cell lymphoproliferation/lymphomagenesis.

miRNA	Expression	Biological material	Disease stage	Proposed biological or clinical relevance	Ref.
**miR-26b**	Reduced	SMZL tissue; PBMCs	HCV-positive SMZL, HCV-MC/CV and HCV-associated NHL	Candidate tumour suppressor, marker of HCV-driven B-cell expansion; levels may normalize after MC/CV remission.	[104, 108]
**miR-30b**	Increased	FFPE lymphoma tissue	HCV-positive indolent B-cell NHL	HCV-associated tissue miRNA signature reflecting an infection-related imprint on indolent lymphoma biology.	[105]
**miR-223**	Altered; HCV-associated	FFPE lymphoma tissue	HCV-positive nodal MZL	Exploratory, histology-restricted association with HCV infection.	[105]
**miR-29a miR-29b**	Altered; HCV-associated	FFPE lymphoma tissue	HCV-positive nodal MZL	Exploratory subtype-specific component of the HCV-associated miRNA profile.	[105]
**miR-21**	Increased	PBMCs	HCV-associated NHL	NHL-restricted peripheral-blood signature and candidate marker of progression from benign lymphoproliferation to overt malignancy.	[108]
**miR-16**	Increased	PBMCs	HCV-associated NHL	Component of a lymphoma-associated miRNA signature; less consistently altered in the pre-lymphomatous stage.	[108, 109]
**miR-155**	Increased	PBMCs	HCV-associated NHL	Oncogenic miRNA associated with B-cell activation and a lymphoma-restricted signature.	[108, 109]
**miR-17-92 cluster**	Dysregulated/increased	PBMCs	HCV-related CV	Associated with increased c-MYC expression, suggesting activation of oncogenic circuits in pre-lymphomatous phase.	[107]
**miR-138-5p**	Reduced	DLBCL tissue	HCV-associated DLBCL	Part of the HCV-DLBCL miRNA profile and associated with clinical outcome.	[106]
**miR-147a miR-147b**	Increased	DLBCL tissue	HCV-associated DLBCL	Associated with clinical outcome.	[106]
**miR-511-5p**	Increased	DLBCL tissue	HCV-associated DLBCL	Associated with clinical outcome.	[106]
**exo-miR-122**	HCV-induced/enriched	Serum- or hepatocyte-derived exosomes	Chronic HCV infection and early immune activation	Activates TLR7–NF-κB signalling in macrophage-lineage cells and promotes BAFF production.	[110]
**exo-let-7b**	HCV-induced/enriched	Serum- or hepatocyte-derived exosomes	Chronic HCV infection and early immune activation	Promotes TLR7-dependent macrophage activation and BAFF production.	[110]
**exo-miR-206**	HCV-induced/enriched	Serum- or hepatocyte-derived exosomes	Chronic HCV infection and early immune activation	Promotes TLR7–NF-κB-mediated BAFF production.	[110]

Supplementary Table 1. The table summarizes miRNA alterations detected in lymphoma tissues, peripheral blood mononuclear cells, and circulating or hepatocyte-derived exosomes across the continuum from chronic HCV infection and mixed cryoglobulinemia/cryoglobulinemic vasculitis to overt B-cell non-Hodgkin lymphoma. Expression changes are relative to the comparator groups used in the corresponding studies. Several findings, particularly subtype-specific associations and prognostic correlations, were derived from relatively small cohorts and should therefore be considered exploratory. MC, mixed cryoglobulinemia; CV, cryoglobulinemic vasculitis; NHL, non-Hodgkin lymphoma; SMZL, splenic marginal zone lymphoma; DLBCL, diffuse large B-cell lymphoma; PBMCs, peripheral blood mononuclear cells; FFPE, formalin-fixed paraffin-embedded; BAFF, B-cell activating factor.
